# Advances in Microbial Remediation of Heavy Metal-Contaminated Soils: Mechanisms, Synergistic Technologies, Field Applications and Future Perspectives

**DOI:** 10.3390/toxics13121069

**Published:** 2025-12-12

**Authors:** Hongxia Li, Xinglan Cui, Yingchun Sun, Peng Zheng, Lei Wang, Xinyue Shi

**Affiliations:** 1 National Engineering Research Center for Environment-Friendly Metallurgy in Producing Premium Non-Ferrous Metals, GRINM Resources and Environmental Technology Corporation Limited, Beijing 101407, China; lihongxia@grinm.com (H.L.); 15966001661@163.com (Y.S.); wanglei@grinm.com (L.W.); shixinyue@grinm.com (X.S.); 2 CECEP DADI (Hangzhou) Environmental Remediation Corporation Limited, Hangzhou 310013, China; hjgczhengp@163.com

**Keywords:** heavy metal contamination, microbial remediation, mechanisms, synergistic technology, field application

## Abstract

Heavy-metal contamination poses a significant global threat to soil environments, underscoring the necessity for effective and sustainable remediation technologies. This review methodically summarizes advances in the field of microbial remediation of heavy metal-contaminated soils, organized around four major dimensions: remediation mechanisms, synergistic technologies, field applications, and future prospects. Firstly, the remediation mechanisms are elucidated, encompassing molecular interactions, cellular adaptation, and community-level cooperative responses. Secondly, the integration of microbes with functional materials and bioelectrochemical systems (BESs) is evaluated, with these materials providing support, electron mediation, and micro-environment regulation that markedly improve remediation efficiency and stability. Moreover, illustrative field cases demonstrate pivotal technological pathways and cost-effectiveness when transitioning from laboratory- to field-scale applications. Finally, emerging frontiers such as synthetic biology-engineered microbes, AI-driven microbial design, circular-economy value recovery, and policy-governance innovations are discussed, proposing essential elements for building a “predictable-controllable-sustainable” microbial remediation platform. This review aims to provide a comprehensive knowledge framework for researchers and to offer decision-making guidance for practitioners and policymakers, thereby advancing microbial remediation toward higher efficiency, reliability, and scalability.

## 1. Introduction

Heavy metal contamination in soil poses a persistent and complex global challenge, affecting ecological health, agricultural productivity, and socio-economic resilience. Intensive anthropogenic activities (e.g., mining, smelting, electroplating, and the excessive application of agrochemicals) have contributed to the accumulation of heavy metals in soil [1,2]. Unlike organic pollutants, heavy metals are inherently non-biodegradable, allowing them to persist in soil for decades or centuries. The long-term existence results in chronic ecological exposure, bioaccumulation in food chains, and human health risks such as carcinogenicity, neurotoxicity, and endocrine disruption [3]. Recent surveys reported that approximately 14–17% of the world’s cultivated land has been contaminated by arsenic, cadmium, cobalt and other heavy metals [4]. In China, the proportion of monitored soil sites exceeding national contamination thresholds has reached 16.1%, with inorganic pollutants contributing to over 80% [5].

Conventional remediation technologies such as chemical leaching, stabilization, and solidification have been widely employed for on-site treatment [6]. However, these conventional methods exhibit inherent drawbacks that have been extensively documented in both research and field applications. Chemical leaching, for instance, often generates secondary pollution of metal-laden wastewater that requires costly secondary treatment [7]. While solidification can reduce the mobility of heavy metals, it may irreversibly alter soil structure and preclude future agricultural use. Furthermore, the substantial chemical and energy inputs associated with these techniques pose significant challenges to their long-term economic and environmental sustainability. Consequently, despite decades of technical refinement, the “treatment–recontamination” cycle remains a persistent issue, with remediation expenses frequently surpassing the economic value of the remediated land [8].

Microbial remediation has matured over the past three decades as a promising in situ technology for potentially self-sustaining heavy metal immobilization or removal [9]. Unlike conventional physicochemical methods, this method exploits synergistic mechanisms including extracellular precipitation, biosorption, bioaccumulation, redox transformations, and metabolite-mediated chelation [3,10]. These processes are regulated by microbial community dynamics, gene expression patterns, and metabolic interactions with plants/soil organic matter. Early single-strain applications using model organisms (such as *Pseudomonas putida* and *Bacillus subtilis*) demonstrated efficacy in controlled environments but exhibited inconsistent field outcomes due to soil heterogeneity and abiotic stress variability [11]. Recent advances have therefore shifted toward multi-modal integrated systems combining microorganisms with hyperaccumulator plants, engineered biochar, or functional nanomaterials [12]. Such systems enhance remediation efficiency while maintaining functionality under environmental fluctuations, establishing foundational platforms for targeted remediation ecologies.

Significant knowledge gaps persist in mechanistic understanding and practical application of microbial remediation. Mechanistically, molecular interaction networks underlying performance variations remain poorly explained, perpetuating a “black box” perception of microbial remediation. Fragmented data on biosorption kinetics and gene expression profiles limit generalizable framework development. Field-scale studies are critically deficient, with scarcely conducted longitudinal monitoring (>3 years), preventing robust assessment of long-term stability and climate resilience. For example, a relatively long-term study of heavy metal contaminated landfill soil demonstrated that applications of filamentous fungi consortia reduced heavy metals by 42–62% after remediation of 144 days [13]. As a whole, the absence of integrated datasets spanning molecular mechanisms to ecosystem-scale outcomes obstructs predictive model development for site-specific remediation. Furthermore, emerging field intersections such as CRISPR-enhanced microbial strains and machine learning models involving microbial remediation remain underexplored [14].

These gaps motivate a systematic synthesis of the microbial remediation research that integrates molecular mechanisms, engineering innovations, and field application into a coherent framework. In this review, we address this need by combining bibliometric mapping with a multi-scale analysis of microbial remediation strategies for heavy metal-contaminated soils. Through analysis of publication trends, keyword co-occurrence networks, and thematic clusters, this paper identifies how research focus has shifted over the past three decades and how emerging directions are reshaping both scientific foundations and application frontiers. This review aims to provide a clear roadmap for researchers while offering a reference for practitioners and policymakers seeking to translate laboratory insights into scalable, sustainable remediation systems.

## 2. Knowledge Landscape: A Bibliometric Overview

To trace the evolution of the field of microbial remediation for heavy metals over the past three decades, we conducted a targeted literature search in the Web of Science Core Collection, including SCI-E, SSCI, and CPCI sub-databases. A compound search string, (“heavy metal” OR “metal contamination”) AND (“microbial remediation” OR “bioaugmentation” OR “bioremediation” OR “microbe-assisted phytoremediation”), was designed to account for terminological variations in literature. The time span was set from 1990 to 2025 to reflect both foundational studies and recent advances, and 1200 records were obtained. The top 20 frequently cited or mentioned terms were selected as keywords. Weakly related literature is filtered out after evaluation to mitigate interference in analysis. Finally, 186 publications were selected and formed the analytical core of this review.

**Figure 1:**
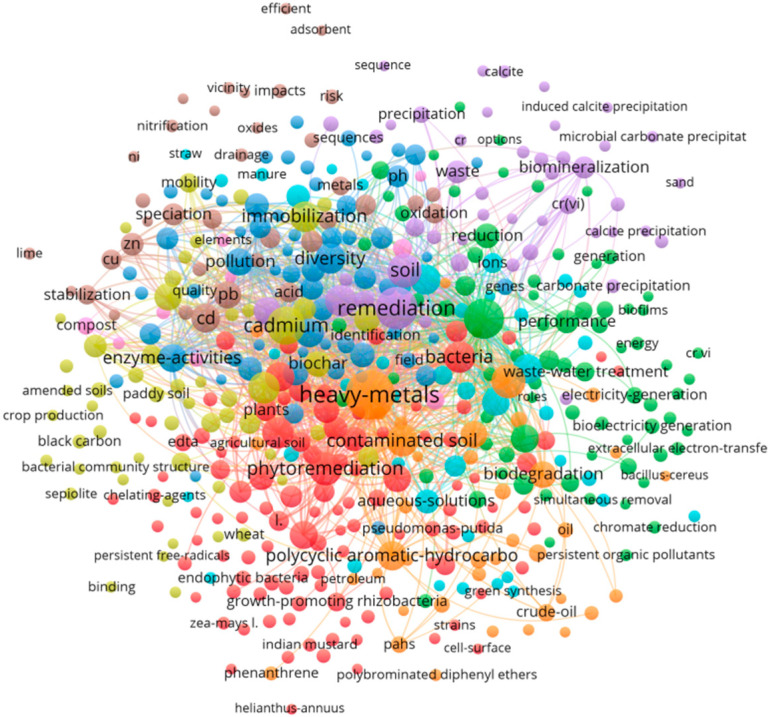
Bibliometric network map (1990–2025 hotspot evolution).

Bibliometric analysis was conducted using VOSviewer (v1.6.18) and CiteSpace (v6.3.R3) to visualize co-occurrence networks, identify keyword clusters, and track temporal shifts in research focus. As illustrated in Figure 1, the research in this field is primarily divided into three main domains: (1) remediation mechanisms (e.g., biomineralization, precipitation, oxidation, reduction, enzyme-activities, extracellular electron-transfer, and gene), (2) integrated technologies (e.g., phytoremediation, biochar, black carbon, and sepiolite), and (3) field applications (e.g., field, paddy soil, and crop production). These bibliometric clusters not only map the evolution of research hotspots but also form the organizing framework for Section 3, Section 4, Section 5 and Section 6, where each cluster is expanded into a dedicated thematic axis covering mechanisms, synergistic technologies, field applications, and emerging frontiers. Thus, this analysis supports to integrate quantitative trends of literature reviews with remediation mechanism and field insights.

## 3. Microbial Remediation Mechanisms: From Molecules to Communities

### 3.1. Molecular Interactions: Biosorption and Redox Transformation

**Figure 2:**
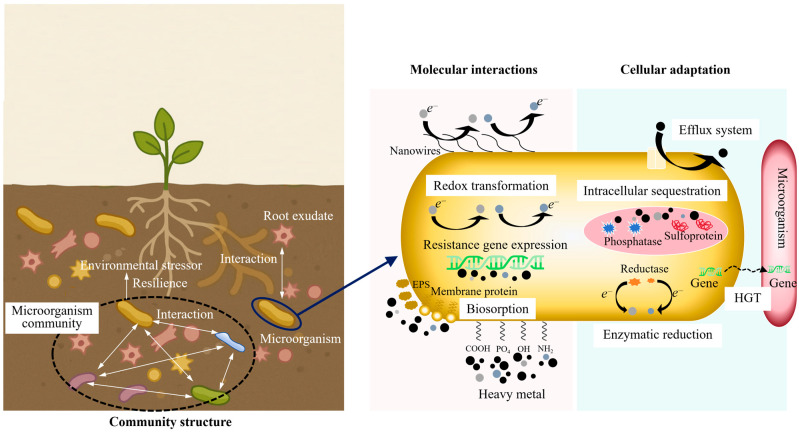
Multi-scale mechanisms of microbial remediation of heavy metals.

The initial interaction between microbial cells and heavy metal ions is generally an active process that begins at the cell surface. As shown in Figure 2, at this interface, a dense and chemically heterogeneous layer composed of functional groups (such as carboxyl, phosphoryl, hydroxyl, and amino residues) interact directly with hydrated metal cations [15]. These functional groups are not randomly distributed across the cell surface; rather, their organization is structurally determined. For instance, Gram-positive bacteria possess a thick peptidoglycan matrix with embedded teichoic acids. This structure forms a high-density ligand environment. It can rapidly immobilize divalent and trivalent metal ions upon contact [16]. In contrast, Gram-negative bacteria predominantly utilize outer membrane proteins, porins, and extracellular polymeric substances (EPSs) to provide binding sites and modulate the diffusion kinetics of metal ions. These inherent structural distinctions account for the considerable variation in biosorption efficiency observed among microbial strains with comparable metabolic capabilities.

Over the past decade, this fundamental model of cell surface sorption has undergone deliberate modifications in laboratory settings. One of the most notable advancements has been the development of engineered surface chemistries aimed at promoting the biosynthesis of phosphomelanin, which is a phosphate-rich pigment formed through tyrosinase-mediated oxidative polymerization of tyrosine derivatives [17]. Phosphomelanin-producing bacterial strains have consistently outperformed conventional EPS-rich strains, demonstrating sorption capacities for Cr(Ⅲ) and other high-valence metals, that surpass even well-characterized melanin analogs. This enhanced performance is due to the formation of stable P-O-C diester linkages between phosphate groups and metal ions. These linkages create coordination complexes. The complexes remain intact under fluctuating pH conditions or high ionic strength, typically these factors compromise weaker chelation systems. In practical applications, such stability could enable long-term metal immobilization in environments with variable climatic conditions or in soils subjected to seasonal flooding, which are rarely achieved by purely natural biosorption mechanisms.

A distinct but increasingly significant interaction mechanism involves cation–π bonding. Far from being a mere chemical curiosity, this phenomenon plays a crucial role in co-contaminated soils where heavy metals coexist with hydrophobic organic pollutants such as polycyclic aromatic hydrocarbons (PAHs) [18]. Microbial metabolites (including phenazines, quinones, and certain indole derivatives) can facilitate the formation of Cd^2+^-PAH complexes at the microbe–soil interface. Notably, while such complexation may reduce the immediate bioavailability of cadmium, it can also, in some cases, intensify oxidative stress within microbial cells by inducing reactive oxygen species (ROS) production. This dual impact has important implications for remediation system design, as failure to account for these indirect pathways may lead to misinterpretation of metal bioavailability data or underestimation of stress-induced declines in microbial activity.

Microbial redox transformation continues to be one of the most extensively studied and most dependable detoxification mechanisms. For heavy metals such as chromium, mercury, and uranium, enzymatic processes catalyze the conversion of highly soluble, toxic species into less soluble forms via chromate reductases, mercuric reductases, and multiheme cytochromes, respectively [19]. However, not all electron transfer processes are enzyme-mediated. Species such as *Geobacter* and *Shewanella* use conductive pili and extracellular nanowires to transport electrons over micrometer-scale distances, enabling metal reduction in locations physically separated from metabolically active cells [20]. This spatial separation may be particularly advantageous in heterogeneous soils, where physical barriers such as pore structures or mineral coatings hinder direct microbial–metal contact. In pilot-scale BES, such long-range extracellular electron transfer (EET) has been shown to accelerate U(VI) reduction and enhance Hg(II) immobilization stability, although the energy efficiency and ecological robustness of these mechanisms remain insufficiently explored [21].

Compared to abiotic absorbents such as activated carbon, synthetic clays, or ion-exchange resins, microbial systems offer the unique advantage of metabolic adaptability. A living sorbent can respond to nutrient availability, modulate gene expression under stress, and even undergo evolutionary adaptation under selective pressure. However, this adaptability is not unconditional. It is influenced by redox gradients, competing cations, and shifts in the microbial energy budget. These constraints are particularly relevant for engineered strains, as synthetic pathways often impose significant metabolic burdens [19]. When deployed in nutrient-poor or highly oxidizing soils, such strains may experience a sharp decline in biosynthetic output regardless of their initial design. For instance, we noted that while microbial strains such as *Geobacter* can achieve over 90% reduction of Cr(VI) under controlled lab settings, their efficiency often drops to approximately 60% in field trials due to soil heterogeneity, competing microbial populations, and fluctuating environmental conditions [22]. Therefore, successful field deployment requires not only a thorough mechanistic understanding of metal binding and transformation processes but also a realistic assessment of the environmental constraints.

Different microbial remediation mechanisms vary markedly in efficiency, stability, and environmental requirements. While biosorption is rapid, it is susceptible to environmental chemical conditions and may lead to the re-release of heavy metals. In contrast, biosolubilization and redox transformation can more permanently immobilize heavy metals. However, the former often relies on specific microbial metabolic activities and soil chemical conditions (such as pH and redox potential), while the efficiency of the latter relies on specific metabolic conditions by the availability of electron donors/acceptors. These mechanisms rarely occur in isolation in natural environments, and they typically synergize within microbial communities. For example, sulfate-reducing bacteria (SRB) can generate S^2−^ through reduction, which then forms stable sulfide precipitates with metal ions. Consequently, effective remediation requires strategic selection or combination of mechanisms based on contaminant type, concentration, and site-specific geochemical conditions.

### 3.2. Cellular Adaptation: Resistance, Detoxification, and Gene Transfer

At the cellular level, microorganisms deploy a diverse of resistance and detoxification mechanisms to survive under high heavy metal concentrations. These mechanisms have been shaped by long-term evolutionary exposure to both naturally occurring (geogenic) and human-induced (anthropogenic) contaminants. Among the most extensively studied mechanisms are active efflux systems, intracellular sequestration, enzymatic reduction or transformation of toxic species, and the formation of biofilms that serve as communal protective structures.

Efflux pumps (particularly P-type ATPases, cation diffusion facilitators, and members of the resistance–nodulation–cell division transporter family) usually function as the first line of defense, actively transporting metal ions from the cytoplasm to the intermembrane space or the extracellular space [23]. Their activity is precisely regulated by metal-responsive regulatory circuits, typically consisting of metal-responsive transcriptional regulators encoded within operons. This regulatory structure enables cells to initiate resistance responses almost immediately upon detecting an influx of toxic ions. However, this rapid responsiveness entails metabolic trade-offs. In nutrient-limited environments, where energy conservation is critical, the operation of high-capacity efflux systems can deplete ATP reserves and divert metabolic resources away from essential processes such as growth, sporulation, or competitive colonization.

Intracellular sequestration represents an alternative yet complementary defense mechanism [15]. In many microbial species, metallothioneins bind and neutralize excess metal ions, while polyphosphates and specialized inclusion bodies act as intracellular storage compartments [24]. These mechanisms are highly effective in buffering against sudden increases in metal concentrations. However, their capacity is inherently limited. Once binding sites are saturated, intracellular metal levels can rise to toxic levels. Furthermore, upon cell death or lysis, sequestered metals may be released back into the environment, potentially reversing the immobilization effect. Whether microbial sequestration leads to long-term environmental stabilization or merely delays metal release remains an open question with significant implications for large-scale remediation strategies.

An emerging area of research focuses on how heavy metal stress influences microbial genome dynamics and promotes horizontal gene transfer (HGT). Mobile genetic elements (including plasmids, transposons, and integrons) frequently carry metal resistance operons and can be transferred not only between closely related bacterial strains but also across broader taxonomic boundaries [25]. Metagenomic studies of heavily contaminated soils have revealed extensive networks of resistance gene exchange, indicating that adaptation to metal stress is not only an individual survival strategy but also a community-level phenomenon. While this genetic fluidity can enhance the resilience and functional diversity of native microbial communities, it also raises biosafety concerns regarding the potential transfer of engineered resistance traits to unintended recipients.

Community-level coordination mechanisms add further complexity. Quorum sensing systems, which regulate biofilm formation, nutrient sharing, and cooperative metabolic activities, are often disrupted by elevated metal concentrations [26,27]. Certain metal ions can interfere with autoinducer synthesis or signal reception, leading to the disintegration of previously stable microbial consortia and impairing processes, such as syntrophic degradation or coupled redox cycling. For remediation systems that rely on such cooperative behaviors, disruption of regulated functions can significantly reduce treatment performance. On the other hand, there is increasing interest in harnessing and modulating sensing to improve spatial organization and enhance resistance in designed microbial consortia, effectively transforming a vulnerability into a strategic advantage.

Although these resistance mechanisms are well-documented in model organisms such as *Escherichia coli* and *Pseudomonas putida*, their expression and effectiveness in native soil microorganisms remain poorly understood [23]. Field-derived communities frequently contain resistance genes whose functions are predicted but not experimentally validated, and their phenotypic expression is frequently influenced by local geochemical conditions, organic matter availability, and interspecies competition. Integrating metagenomic and transcriptomic data with process-based kinetic modeling offers a promising approach to bridge the gap between genetic potential and actual ecosystem function in the context of heavy metal remediation.

### 3.3. Community Structure and Rhizosphere Effects

At the community level, the efficacy of microbial remediation is influenced not only by the inherent capabilities of individual strains but also significantly by the broader ecological context. This interaction is particularly evident in the rhizosphere, which is the narrow, chemically and biologically dynamic zone surrounding plant roots. Within this interface, root exudates (such as organic acids, flavonoids, and various signaling molecules) continuously shape the microbial community structure [28]. Rather than serving merely as general nutrient sources, these exudates function as ecological filters. For instance, secondary metabolites like luteolin and coumarins can selectively promote the growth of metal-tolerant taxa (such as *Pseudomonas* spp.), which in turn contribute to soil detoxification and enhance plant growth under stressful conditions. In practical applications, these targeted plant–microbe interactions have been harnessed in phytoremediation strategies, where co-inoculation with plant growth-promoting *Rhizobacteria* has improved both metal immobilization and biomass recovery, often alleviating the physiological burden on the host plant [29]. Fungal networks further enrich this ecological framework. Arbuscular mycorrhizal fungi (AMF, such as *Rhizophagus irregularis* and *Funneliformis mosseae*) extend their hyphae well beyond the immediate rhizosphere, accessing nutrients and mitigating metal stress in regions that root hairs cannot reach [30]. In soils affected by multiple contaminants or nutrient depletion, AMF often act as both structural supports and biochemical facilitators, harboring diverse bacterial communities on their hyphal surfaces [31]. These symbiotic associations are not static. They facilitate metabolite diffusion and chemical signaling, thereby stabilizing microbial community dynamics under variable redox or moisture conditions.

The composition and structure of the microbial community itself play a crucial role in the stability of remediation effect. Communities featuring multiple taxa performing similar detoxification functions, though exhibiting high functional redundancy, are generally more resilient to environmental disturbances [32]. In diverse microbial communities, one organism’s metabolic byproducts become another’s nutrients. This cross-feeding sustains metal transformation efficiency, even as environmental conditions shift. In contrast, low-diversity inoculants are far less resilient. Monocultures, in particular, can collapse abruptly when faced with stressors like sudden pH changes or competition from native microbes. Many commercial bioaugmentation products still depend on a handful of standardized strains. Often, they are deployed with little regard for their ecological compatibility in the target soil. Although monoculture inoculants are often criticized for their limited resilience, an important nuance lies in the so-called priming effect, the introduction of an external microbial strain can stimulate the metabolism of indigenous soil communities, leading to broader shifts in enzyme activity, redox conditions, and carbon turnover. This mechanism partly explains why single-strain inoculants can produce short-term benefits even when they do not persist over time. However, such priming tends to be transient. Many low-diversity inoculants eventually lose efficacy under abrupt pH shifts, salinity fluctuations, or competitive pressure from resident soil taxa [33].

Field observations continue to highlight the risks associated with such ecological mismatches. Introduced microbial strains may fail to establish, be outcompeted by native species, or integrate poorly into local metabolic networks, resulting in diminished performance beyond controlled experimental settings. To mitigate these challenges, the concept of synthetic microbial communities (SynComs) has gained increasing attention. SynComs are engineered consortia with defined compositions and modular functionalities, designed to merge the resilience of natural communities with the predictability of engineered systems. However, post-deployment stability remains a significant hurdle. Without strategies to preserve spatial organization, mutualistic interactions, and adaptive responses to environmental signals, SynComs may degrade into less effective microbial community over time.

Advancing this field requires integrating mechanistic insights across multiple scales, from molecular interactions at the cell surface to the emergent properties of entire microbial networks. Achieving this will necessitate a coordinated approach that combines omics-based profiling, ecological modeling, and long-term field trials, rather than relying solely on laboratory-scale experiments. Only through such a multiscale integration can microbial remediation evolve from a promising concept into a reliable, context-aware technology capable of addressing heavy metal contamination across the complexity of real-world soil environments.

## 4. Synergistic Technologies: Enhancement of Microbial Remediation

### 4.1. Microbial Genetic Engineering

**Figure 3:**
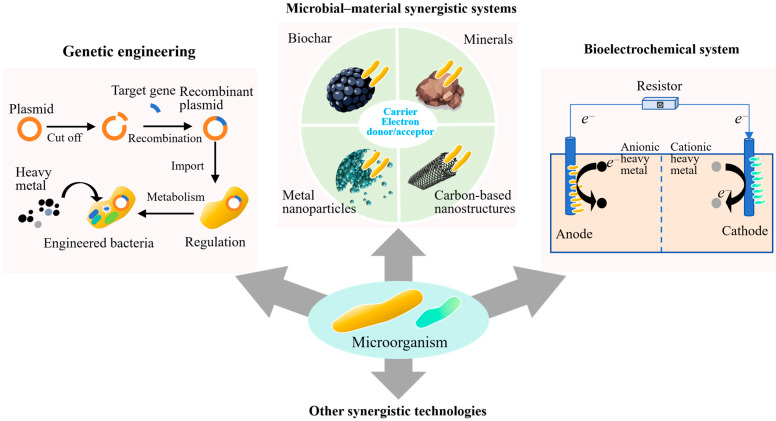
Synergistic Technologies for microbial remediation.

As shown in Figure 3, genetic engineering is increasingly recognized as a viable strategy for overcoming the limitations of unmodified microbial systems in heavy metal-contaminated soils [15]. Native strains often exhibit a certain level of metal tolerance. However, their metal removal efficiency can be highly variable. This is because it is influenced by environmental factors such as pH, ionic strength, and nutrient availability. Moreover, few naturally isolated strains possess metabolic pathways that are simultaneously optimized for both metal resistance and catalytic transformation. To address these limitations, synthetic biology approaches are being employed to re-engineer these traits with greater precision and specificity, leveraging modular genetic tools to enhance biosorption capacity, redox activity, and environmental resilience.

A notable advancement in this field is the incorporation of metal-binding proteins and pigment biosynthesis pathways into robust microbial hosts. For example, *Bacillus subtilis* strains engineered to express tyrosinase have demonstrated significantly higher production of phosphomelanin compared to naturally pigmented strains [17]. This phosphate-rich pigment forms stable P-O-C diester bonds with trivalent metal ions, the binding mechanism of which remains effective under acidic conditions and across a wide range of ionic environments. Comparative studies with natural strains have shown that these engineered strains not only achieve greater removal efficiencies for heavy metals but also maintain activity over extended exposure periods and exhibit reduced sensitivity to pH fluctuations. Furthermore, genetic engineering has enabled the integration of multiple functional traits within a single microbial host. Some engineered strains of *Escherichia coli*, for instance, have been designed to co-express heavy metal chelators and enzymes (such as polyethylene terephthalate hydrolases), enabling simultaneous polymer degradation and metal immobilization [34]. While such dual-function systems offer significant potential for treating complex contaminants, they also impose a measurable metabolic burden on the host. Under carbon-limited rhizosphere conditions, the energy demand for maintaining both functions can account for over 40% of available ATP, thereby reducing the competitiveness of engineered strains against indigenous microbial populations [35].

A persistent challenge has been the gap between laboratory performance and real-world applicability. Strains that perform well under controlled conditions often fail to maintain functional dominance in natural soil environments, where they may be outcompeted by native taxa better adapted to local ecological pressures. This issue has prompted research into genetic containment and stability strategies, including inducible suicide circuits that respond to environmental triggers such as light exposure or pH changes, as well as synthetic auxotrophy-deficiency technology that renders microbial growth dependent on nutrients available only in laboratory settings [36]. Although these approaches help mitigate risks related to HGT and ecological disturbance, few have been rigorously tested in long-term field application, with most evaluations still conducted in controlled mesocosm systems.

Recent advances in CRISPR interference (CRISPRi) and recombineering technologies have enabled more precise regulation of gene expression, allowing engineered microbes to dynamically adjust their metabolic activity in response to environmental signals rather than maintaining constitutive high-level expression [37]. Furthermore, genome-scale metabolic modeling is increasingly used to design microbial hosts that effectively balance redox potential, energy allocation, and cofactor requirements, thereby minimizing the fitness costs associated with engineered traits. As regulatory frameworks continue to evolve, the practical deployment of genetically engineered microbes for environmental remediation will depend not only on their technical performance but also on their demonstrated safety, controllability, and ability to function autonomously without continuous external input.

Performance comparisons across engineered microbial platforms reveal clear trade-offs. Tyrosinase-expressing *Bacillus* strains often achieve the highest sorption capacities under acidic or multi-metal conditions, whereas engineered *E. coli* chassis provide superior genetic controllability but struggle with environmental persistence. Some studies report enhanced Cr(VI) reduction with engineered reductase-overexpressing strains, while others note steep declines in activity after exposure to fluctuating moisture or competition from native taxa [14]. These conflicting outcomes underscore the need to evaluate engineered strains not only by their intrinsic biochemical capacity but also by their ecological compatibility.

### 4.2. Microbial–Material Synergistic Systems

Integrating microbial systems with functional materials has emerged as an effective strategy for enhancing the stability, persistence, and catalytic performance of bioremediation processes in heavy metal-contaminated soils. Functional materials (e.g., biochar, metal oxide nanoparticles, minerals, and carbon-based nanostructures) serve not only as inert carriers but also as active components, functioning as immobilization supports, redox cofactors, and microenvironment regulators [38,39]. Among these, biochar–microbe composites have attracted considerable attention. This is due to their compatibility with many microbial species, high surface area, and capacity to adsorb and buffer contaminants [40]. Field-oriented studies have demonstrated that biochar derived from agricultural residues (such as corn straw or bamboo) can effectively support fungal inoculants like *Trichoderma harzianum*, resulting in reductions in the exchangeable fractions of heavy metals. Application of 5% rice straw biochar can decrease the availability of heavy metals to microbes by increasing organic-bound fraction of heavy metals by up to 68% [38]. Comparative analyses consistently indicate that these composites outperform free-cell inoculations, primarily due to improved colonization stability, enhanced water retention, and increased resistance to pH fluctuations and transient metal exposure.

Nanomaterial-assisted systems provide similar benefits with achieving 1–2 times higher heavy metal removal efficiency albeit through distinct mechanisms [41]. Oxide nanoparticles such as ZnO, Fe_3_O_4_, and TiO_2_ can directly modulate microbial metabolism by influencing enzyme activity or facilitating electron transfer [37,42,43]. For example, ZnO nanoparticles have been shown to enhance the expression of Pb-reducing enzymes in *Bacillus haynesii*, significantly accelerating remediation under controlled conditions. However, these effects are highly concentration-dependent, moderate concentrations can stimulate microbial activity, whereas higher doses (>1 mg/kg) often induce oxidative stress or membrane disruption [43,44]. The exact mechanisms driving the observed synergistic effects remain under debate. Some studies suggest electron shuttling or catalytic surface reactions mediated by nanoparticles, while others attribute the effects to stress-induced activation of microbial detoxification pathways. More advanced hybrid systems integrate biochar with nanoparticles, integrating the adsorptive and habitat-supporting properties of biochar with the catalytic or coprecipitation capabilities of nanomaterials. For instance, Fe nanoparticle-impregnated biochar has been employed to immobilize arsenic while promoting the growth of arsenite-oxidizing bacteria. Despite their potential, these systems encounter practical limitations, such as particle aggregation may reduce reactivity, metal leaching from the carrier can pose secondary contamination risks, and performance may be compromised under waterlogged or alkaline soil conditions [45]. Nonetheless, material–microbe composites offer notable benefits. Coupling microorganisms with other technologies can significantly overcome numerous limitations inherent to single microbial systems. However, each synergistic technique has specific application scenarios and constraints. For instance, microbial–plant remediation is ecologically friendly but involves lengthy remediation cycles and may be constrained by plant biomass. Microbial–biochar composites excel at enhancing microbial survival rates and soil improvement, yet their long-term stability and universality across different soils require further field validation. Although microbial–nanomaterial systems demonstrate highly efficient performance in laboratory settings, their high costs and potential ecological safety risks represent bottlenecks that must be addressed before field deployment. Consequently, future research should focus on developing site-specific, intelligently responsive synergistic systems while balancing their environmental benefits against potential risks within life cycle assessment (LCA) framework. Yet, variability in performance across different soil types and seasons remains a concern, and production costs (particularly for functionalized nanomaterials) continue to impede large-scale implementation.

When customized to site-specific conditions, material–microbe systems exhibit high adaptability. Their composition can be precisely engineered to target specific contaminants or to match local soil chemistries and climatic conditions. A promising future direction lies in the development of integrated material–microbe responsive systems in which material properties dynamically adjust in response to microbial metabolic activity or environmental stimuli. For instance, biochar–microbe systems that modulate surface charge achieve pH-responsivity, thereby enabling self-regulating and adaptive remediation under field-scale conditions.

### 4.3. Bioelectrochemical Systems for Directed Remediation

The BESs offer a promising approach to microbial remediation by enabling precise control over redox processes, which conventional systems cannot achieve. In their simplest form, BESs combine electroactive microorganisms with solid-state electrodes embedded in contaminated soils, enabling efficient electron transfer between microbial pathways and pollutants. This direct exchange supports key reactions such as methylmercury demethylation, Cr(VI) reduction to Cr(III), and immobilization of arsenic or uranium through reductive precipitation [46]. Unlike suspended-culture or ex situ systems, soil-based BESs operate in situ and can be designed to create localized redox gradients that promote specific microbial activities [47]. By adjusting electrode placement and voltage, zones can be formed to support iron-reducing, sulfate-reducing, or methanogenic communities, each aligned with the remediation goal. This approach works well in soils where natural redox conditions vary due to seasonal water levels or uneven drainage.

Electrogenic bacteria like *Geobacter* and *Shewanella* are key to BESs due to their conductive biofilms and ability to transfer electrons via outer-membrane cytochromes and pili [47]. Studies show that BESs using these strains remove contaminants faster than passive systems. However, their performance in natural soils is limited by low moisture, poor buffering, and competition from non-electrogenic microbes. These issues are more severe in coarse soils where electrode–microbe contact is inconsistent. Recent electrode improvements aim to overcome these issues. Materials like graphite–graphene composites, conductive polymers, and metal–organic framework coatings increase surface area, improve wetting, and enhance microbial attachment [48,49]. Some also act as electron reservoirs, supporting stable redox cycling under fluctuating current. When paired with genetically engineered electrogens designed for better redox shuttles or cytochromes, these electrodes significantly boost reaction rates.

Despite these advancements, the transition of BESs from laboratory to field-scale application remains challenging. The infrastructure required for electrode installation, external power supply, and wiring can be cost-prohibitive, particularly in large agricultural fields or forested areas [50]. To date, most successful implementations have been limited to small-scale constructed wetlands or shallow subsurface environments, where deployment is less complex. Even in these settings, operational effectiveness relies on precise voltage regulation, appropriate electrode placement, and controlled carbon delivery to avoid stimulating undesirable microbial processes. A promising avenue for future development lies in the integration of BESs with real-time environmental monitoring and adaptive control systems. The incorporation of sensors capable of measuring redox potential, contaminant concentrations, and microbial activity could enable automated adjustments to electrode voltage, nutrient dosing, or inoculum composition. Such self-regulating BES would be capable of responding dynamically to environmental fluctuations without requiring continuous manual intervention, thereby advancing the system toward autonomous and long-term remediation solutions. Realizing this vision will necessitate interdisciplinary collaboration spanning microbial ecology, electrochemistry, and systems engineering, yet it presents a valuable opportunity to merge the precision of electrochemical control with the adaptability of microbial communities. 

## 5. Field Applications: Scaling Challenges and Solutions

### 5.1. Case Studies: From Laboratory to Field Application

Although microbial remediation has been extensively validated in laboratory, its field-scale application remains constrained by poor microbial colonization, reduced metabolic activity under environmental fluctuations, low bioavailability of aged metals, and physical failures of functional materials. Nevertheless, well-documented field studies demonstrate that effective scale-up is achievable. This requires system design, microbial formulation, and operational strategies to be aligned with site-specific conditions. A notable example is the large-scale application of facultative SRB for the remediation of cadmium-contaminated paddy soils [51]. Field implementation via irrigation and root-zone injection has consistently reduced cadmium accumulation in rice grains by more than 90% within a single growing season, which exceeds the efficacy of chemical immobilization agents while avoiding the ecological drawbacks of long-term soil replacement or synthetic chelator use.

In perennial cropping systems such as tea plantations, plant–microbe interactions have demonstrated practical effectiveness. Inoculation with plant growth-promoting rhizobacteria, including *Serratia*, *Pseudomonas*, and *Bacillus* species, has enhanced crop resilience and reduced zinc and lead uptake in shoots by 30–40% in mildly contaminated soils, without compromising yield or product quality [52]. These approaches integrate well with existing agricultural practices and provide dual benefits of contaminant reduction and sustained productivity. However, their efficacy declines significantly in multi-metal contaminated or highly acidic soils, where microbial persistence and root colonization are reduced. Compared with chemical leaching or solidification-stabilization methods, microbial remediation is generally slower and less predictable, particularly in time-sensitive urban brownfield redevelopment projects. However, it outperforms chemical alternatives in long-term soil function restoration, biodiversity enhancement, and carbon footprint reduction. It also supports post-remediation land uses such as afforestation, forage cultivation, or ecological buffer zones, which are often incompatible with aggressive chemical interventions.

Recent initiatives have explored the use of microbial consortia for arsenic and chromium immobilization in mining tailings and industrial buffer zones, frequently co-applied with soil conditioners or hydrogel carriers to enhance microbial viability [53]. Although per-hectare costs remain higher than conventional liming or amendment strategies, the cumulative benefits in nutrient retention, biodiversity preservation, and compliance with agricultural safety standards justify their application in high-value or regulated environments. A persistent challenge across large-scale implementations is the absence of long-term monitoring [54]. Few programs maintain systematic post-application surveillance beyond one or two growing seasons, resulting in uncertainties regarding microbial persistence, contaminant stability, and the potential for pollutant rebound. Addressing this gap is essential to establish microbial remediation as a recognized best practice. Field applications illustrate both the scalability and mechanistic diversity of microbial remediation platforms, while highlighting ongoing challenges related to environmental variability and site-specific constraints.

### 5.2. Microbial Environmental Adaptation and Performance Barriers

**Figure 4:**
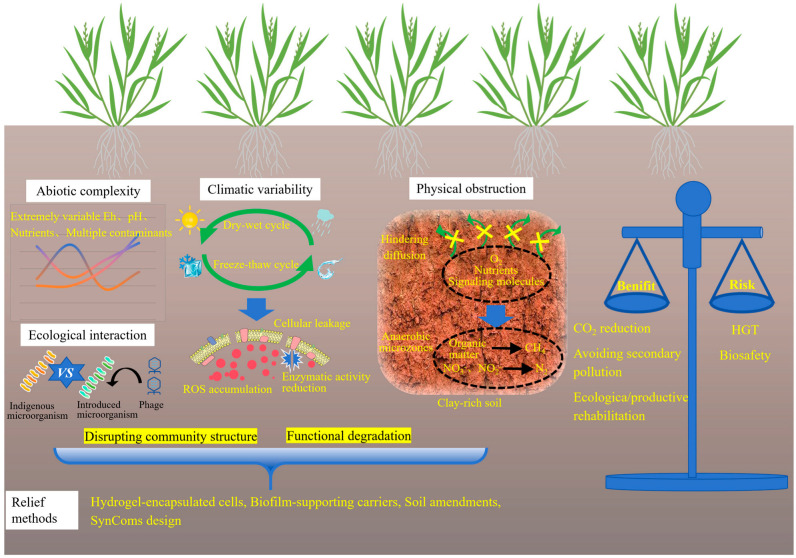
Challenges in the field applications of microbial remediation.

As shown in Figure 4, the primary constraint of microbial environmental adaptation lies in the abiotic complexity of contaminated soils, which includes dynamic redox conditions, extreme pH levels, nutrient scarcity, and the coexistence of multiple contaminants, such as PAHs and residual herbicides [18]. Microbial performance is highly dependent on contaminant bioavailability, which is governed by metal speciation and soil mineral composition. In aged or weathered contamination sites, heavy metals are often sequestered within iron and manganese oxide matrices, significantly reducing their accessibility for microbial transformation. Kinetic studies indicate that metal release rates may be low, rendering microbial remediation ineffective without prior mobilization or pretreatment strategies. While microbial approaches exhibit slower reaction kinetics compared to chemical leaching or surfactant-assisted desorption, they offer superior environmental specificity, and thus a trade-off must be carefully evaluated based on site-specific remediation objectives.

Climatic variability presents an additional challenge. In regions subject to pronounced wet–dry or freeze–thaw cycles, microbial viability and metabolic activity can fluctuate substantially over time. For instance, soil desiccation has been shown to reduce enzymatic activity by more than 40%, primarily due to membrane damage and oxidative stress. Freeze–thaw events may compromise biofilm integrity and induce cellular leakage, thereby disrupting community structure and leading to functional degradation. Salinity represents a substantial constraint on field performance. In coastal, estuarine, and irrigation-affected soils, elevated salt concentrations inhibit microbial respiration, compromise membrane integrity, and impair the activity of metal-transforming enzymes. Consequently, microbial inoculants that perform effectively in upland soils often fail in saline coastal remediation trials. Plant–microbe systems are similarly impaired: salinity diminishes root exudation and weakens microbial recruitment. Exceptions include certain halophytes (such as Salicornia europaea, Spartina alterniflora, and Atriplex halimus) which maintain metabolically active rhizospheres under saline conditions. These species have demonstrated particular promise in co-contaminated (metal and salinity) environments, where they support microbial communities capable of sulfate reduction, metal precipitation, and organic acid secretion. Thus, incorporating halophyte–microbe partnerships into remediation frameworks offers a more viable strategy for saline soils than relying exclusively on conventional crop-associated PGPR strains [55]. Although certain extremotolerant species (such as spore-forming *Bacillus* or psychrotolerant *Pseudomonas*) exhibit notable resilience, their metabolic capabilities are typically confined to basic detoxification mechanisms and lack specialized enzymatic pathways necessary for efficient redox cycling or biosorption. Soil texture and structure further influence microbial efficacy. In compacted or clay-rich soils, the diffusion of oxygen, nutrients, and signaling molecules is severely restricted, thus limiting microbial colonization depth and reducing community diversity [56]. In contrast to sandy or loamy soils, where microbial dispersal and rhizosphere interactions are more favorable, fine-textured soils pose significant physical barriers to inoculant establishment. Furthermore, the presence of clay or alkaline soils can lead to poor water permeability and prolonged waterlogging, thereby promoting the formation of anaerobic microenvironments, which favor the occurrence of undesirable biogeochemical processes such as methanogenesis and denitrification. Efforts to mitigate these limitations have included the use of hydrogel-encapsulated cells, biofilm-supporting carriers, and soil amendments such as biochar or perlite. These auxiliary materials enhance moisture retention, create localized niches for microbial colonization, and improve access to immobilized metals. Despite promising outcomes in mesocosm-scale studies, field-scale application remains constrained by high costs, mechanical fragility, and heterogeneous distribution.

An additional challenge arises from the ecological interaction between indigenous microbial communities and introduced strains. Competitive exclusion, phage predation, and functional incompatibility with indigenous microbial communities often lead to a sharp decline in the abundance of inoculated microbes, even when laboratory assessments indicated high compatibility [57]. This underscores the decisive influence of ecological context on the performance of engineered systems. The development of SynComs tailored to local microbial communities and specific soil properties opens a promising avenue for future research [58]. SynComs development is fundamentally not a mere assembly of strains, but a deliberate construction of microbial consortia with defined roles, trophic dependencies, and stress-response mechanisms, designed to function as integrated units. SynComs are designed to deliver greater stability in field conditions and enhanced resistance against competitive displacement. This is achieved by emulating the functional redundancy and interaction networks of natural microbiomes, while preserving the predictability essential to engineered systems. However, this strategy requires substantial upfront investment in metagenomic sequencing and microbial cultivation.

### 5.3. Risk–Benefit Assessment and Biocontainment Strategies

Assessment of microbial remediation must look beyond removal efficiency. It requires a comprehensive evaluation framework. This framework should integrate factors such as environmental performance, long-term sustainability, and societal acceptability. The LCA provides a rigorous methodological basis for comparative analysis, enabling the quantification of cumulative energy consumption, greenhouse gas emissions, and material flows across all stages of the remediation process. Comparative LCA studies consistently demonstrate that microbial-based approaches achieve 40% to 60% reductions in carbon emissions, eliminate the need for high-temperature treatment, avoid secondary pollution pathways, and simultaneously facilitate ecological or productive remediation of contaminated sites [59].

Despite these advantages, the deployment of engineered microbial strains in environmental applications remains constrained by persistent concerns over HGT. The introduction of resistance genes or synthetic metabolic circuits for remediation purposes carries a tangible risk of genetic exchange with indigenous microbial populations, potentially leading to unpredictable ecological consequences. Although natural gene transfer is an inherent feature of microbial ecosystems, the deliberate release of engineered genetic constructs raises significant biosafety concerns, potentially accelerating or altering the trajectory of HGT [60]. These issues have prompted intensified regulatory scrutiny through international frameworks such as the Cartagena Protocol on Biosafety. To address biosafety risks, a series of biosafety containment strategies have been developed to prevent the unintended persistence and spread of genetically modified microorganisms. These strategies include toxin–antitoxin systems, inducible kill-switch mechanisms, and synthetic auxotrophy dependent on non-native metabolites [61,62]. The reliability and stability of these systems in complex soil environments remain uncertain, particularly under dynamic abiotic factors such as fluctuating temperature and pH, as well as competitive pressures from indigenous microbial communities. Moreover, large-scale microbial mortality may induce cascading ecological effects, such as transient nutrient releases, shifts in community structure, or disruptions to biogeochemical cycles [63]. This underscores the necessity for comprehensive environmental impact assessments prior to field-scale implementation.

Social acceptance and regulatory alignment are equally critical, jointly determining whether microbial remediation technologies can transition from experimental applications to widespread adoption. In multiple jurisdictions, bioremediation projects cannot secure public funding or be incorporated into national environmental remediation plans due to the absence of standardized performance metrics or certification protocols [62]. Elsewhere, precautionary regulations restrict the release of living or genetically modified organisms, even when empirical evidence indicates negligible environmental risk. To address this, emerging pilot programs are investigating market-based instruments (such as bioremediation credit systems) which convert quantifiable environmental outcomes (including carbon sequestration, contaminant degradation, and ecosystem recovery) into tradable credits. Such mechanisms not only improve economic feasibility but also align remediation efforts with broader climate mitigation and biodiversity conservation objectives.

Realizing the full potential of microbial remediation requires coordinated action across scientific, ecological, and policy domains. Technical performance assessments must account for site-specific biogeochemical conditions. Regulatory frameworks must evolve alongside advances in synthetic biology. Long-term monitoring mechanisms should be incorporated from the outset of project design. Given the multidimensional nature of these systems, no single metric—whether removal efficiency, cost-effectiveness, or carbon reduction—can fully capture their overall benefits and associated risks. Consequently, embedding microbial remediation within adaptive, integrated environmental management strategies represents the most viable and sustainable pathway to both amplify its impact and safeguard ecological integrity.

## 6. Future Perspectives: Emerging Frontiers in Microbial Remediation

### 6.1. Synthetic Biology-Enabled Microbial Systems

**Figure 5:**
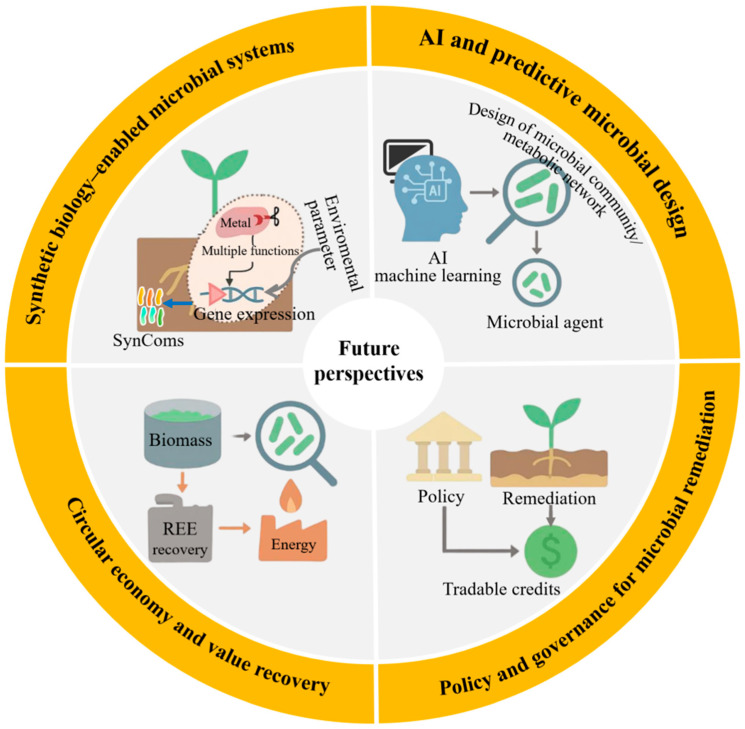
Future perspectives for microbial remediation.

As shown in Figure 5, synthetic biology holds significant potential for reprogramming microbial systems, enabling targeted, controllable, and highly efficient bioremediation [64]. SynComs with complementary metabolic functions have transformed microbial community engineering. Recent studies have demonstrated that CRISPRi-regulated SynComs dynamically allocate metabolic tasks between constituent strains: one optimized for rhizosphere colonization, the other for extracellular phosphatase secretion, thereby enabling targeted metal solubilization without compromising colonization stability. This spatial division of labor, combined with transcriptional logic gates, enables precise regulation of gene expression in response to environmental signals such as pH, redox potential, or metal concentration. Furthermore, synthetic carriers equipped with smart genetic circuits are being developed to operate in a two-stage modes, in which specific environmental triggers activate bioremediation functions after an initial dormant colonization phase [33]. For example, the pDawn-pDark light-responsive kill switch enables externally induced cell lysis upon remediation completion, significantly enhancing ecological biosafety. Similarly, optogenetically regulated toxin–antitoxin systems have been experimentally validated for Cr(VI) remediation applications, where engineered strains achieved >95% reduction of Cr(VI) within 72 h while maintaining strict population control. Despite these advances, synthetic biology-based systems continue to face critical challenges related to long-term genetic stability, regulatory approval pathways, and adaptive trade-offs in real-world environments [64]. Addressing these limitations will require innovations in genetic insulator design, promoters tolerant to field conditions, and in situ monitoring tools capable of tracking synthetic gene expression in complex ecosystems.

### 6.2. Artificial Intelligence and Predictive Microbial Design

The application of artificial intelligence (AI) into microbial remediation research is reshaping the design, optimization, and deployment of microbial community [65]. Machine learning models trained on extensive environmental datasets include soil pH, organic matter content, metal speciation profiles, and indigenous microbial taxa, which can now accurately predict the functional performance of candidate strains under realistic field conditions [66]. These predictive capabilities have enabled the rational design of site-specific microbial agent formulations, allowing inoculants to be precisely tailored to maximize both remediation efficacy and ecological compatibility. Notably, a gradient boosting model trained on over 2000 field datasets achieved 89% accuracy in predicting the potential of *Bacillus* complexes to reduce bioavailability in co-contaminated soils. Beyond strain selection, AI-driven approaches have been applied to metabolic network optimization using genome-scale metabolic models and reinforcement learning to identify genetic targets that enhance metal tolerance and sorption capacity while minimizing metabolic burden [67]. Moreover, the integration of multi-omics data (metagenomics, transcriptomics, metabolomics) into AI frameworks enables real-time system dynamics inference and early warning of operational failures. Nevertheless, the widespread application of AI in bioremediation remains constrained by challenges such as insufficient high-quality data, inconsistent annotation standards, and the absence of centralized, standardized repositories. Overcoming these barriers will necessitate the establishment of federated learning platforms and open-access soil microbiome databases. This will support collaborative model training while safeguarding data privacy, accelerating the adoption of AI-driven microbial design across academia and industry.

### 6.3. Circular Economy and Value Recovery

Integrating microbial remediation into the circular economy framework offers an opportunity to transform environmental liabilities into valuable economic assets. Remediated biomass, typically regarded as waste, can be upgraded through secondary processing methods such as bioleaching, anaerobic digestion, or thermochemical conversion. For instance, microbial biomass generated during rare earth element (REE) recovery from e-waste leachates has been shown to retain more than 80% of adsorbed lanthanum (La^3+^) and cerium (Ce^3+^), achieving subsequent recovery rates exceeding 90% via mild acid desorption [68,69]. Techno-economic assessments indicate that such integrated systems can achieve cost recovery within five years at medium-scale operations, demonstrating economic viability for industrial application. Importantly, coupling remediation with resource recovery enhances system multifunctionality, as demonstrated by dual-purpose technologies that simultaneously remove contaminants and generate renewable energy. Engineered microbial fuel cells inoculated with metal-reducing bacteria have achieved stable power outputs while treating arsenic-contaminated water [70]. Beyond material and energy recovery, the ecological benefits of microbial remediation (such as soil health restoration, carbon sequestration, and biodiversity preservation) can be quantified and monetized through emerging ecosystem service markets. To fully realize this potential, LCA methodologies must be adopted to capture environmental synergies, while policy instruments should be developed to recognize and incentivize non-market value streams.

### 6.4. Policy and Governance for Microbial Remediation

The widespread adoption of microbial remediation technologies hinges on the establishment of supportive policy frameworks and adaptive governance mechanisms. Current environmental regulations frequently lag behind technological advancements, with approval processes for genetically modified microorganisms remaining overly burdensome or undefined in many jurisdictions [71]. A key advancement lies in permitting controlled field trials under transparent, multi-stakeholder oversight, which accelerates evidence generation while safeguarding ecological integrity. Such frameworks can incorporate dynamic risk assessment updated in real time based on monitoring data, thereby improving regulatory responsiveness and public confidence. Beyond regulatory reform, economic incentives (such as bioremediation credit systems) could be integrated into national carbon markets or land-use planning policies. Quantifying carbon sequestration and biodiversity enhancement associated with remediation activities could yield tradable credits within emissions trading schemes or environmental offset programs. Pilot initiatives in the European Union and China have already begun linking microbial remediation projects to measurable outcomes such as soil organic carbon accumulation and nitrogen retention. Furthermore, incorporating microbial solutions into green finance criteria could unlock substantial capital for nature-based remediation enterprises. The green finance criteria include the European taxonomy and Environmental, Social, and Governance (ESG) investment frameworks. To ensure long-term legitimacy and scalability, participatory governance models must be institutionalized throughout project lifecycles, encompassing local communities, industry stakeholders, and environmental agencies. Co-design workshops, open-access data platforms, and citizen science initiatives can foster trust, improve technology alignment with local needs, and enhance social acceptance. When these governance innovations converge with advances in synthetic biology and AI, they pave the way for embedding microbial remediation into pollution control and global sustainability strategies. This integrated approach not only bridges technological innovation with policy evolution but also positions biological systems as active contributors to carbon credit mechanisms and ESG performance indicators.

## 7. Conclusions

This review systematically summarizes the advances in microbial remediation of heavy metal-contaminated soils, covering four major dimensions: mechanistic insights, synergistic technologies, field applications, and future prospects. The main conclusions are as follows:

1. Microorganisms remove or immobilize heavy metals through coordinated processes through cell-surface functional groups, novel metal-binding compounds such as phosphomelanin, and electron-transfer networks. Horizontal transfer of metal-resistance and detoxification genes, together with environmental factor (such as root exudate)-mediated selection and regulation of metal-tolerant microbial consortia, constructs a collaborative ecological network.
2. Integrating microbial systems with functional materials (e.g., biochar, metal-oxide nanoparticles, composite carriers) and bioelectrochemical systems markedly enhances remediation efficiency, prolongs microbial activity, and improves the soil micro-environment. The supportive, electron-mediating, and microenvironment-regulating roles of these materials are key to achieving high-efficiency, sustainable remediation.
3. Research over the past three decades has progressed from single-microbe experiments toward multidisciplinary integration and from laboratory-scale to large-area field deployment. Representative cases (such as SRB in paddy fields and plant growth promoting bacteria in tea plantations) demonstrate the cost-effectiveness and practicability of microbial remediation in real-world engineering.
4. Emerging frontiers, including synthetic biology-engineered microbes, AI-driven microbial design, resource-recovery circular economies, and supportive policy-governance innovations, are expected to shape a future microbial remediation platform characterized by predictability, controllability, and sustainability. Achieving high-efficiency, reliable, and scalable heavy-metal soil remediation will require coordinated advances in microbial functional enhancement, system integration, risk assessment, and standardization.

In summary, microbial remediation, with its low cost, minimal secondary pollution, and ecological friendliness, has become a promising technology for heavy-metal soil remediation. This review provides a comprehensive knowledge framework that deepens mechanistic research for the academic community, offers technical pathways for practitioners, and supplies decision-making guidance for policymakers. Future work focusing on precise microbial functional regulation, interdisciplinary synergistic innovation, and industrialization pathways will propel the field to higher scientific levels and broader application prospects. This, in turn, will make a substantial contribution to the enhancement of soil environmental safety and the promotion of sustainable development.

## Data Availability

All the relevant data are within this manuscript.
